# An Increased Frequency in HLA Class I Alleles and Haplotypes Suggests Genetic Susceptibility to Influenza A (H1N1) 2009 Pandemic: A Case-Control Study

**DOI:** 10.1155/2018/3174868

**Published:** 2018-02-25

**Authors:** Ramcés Falfán-Valencia, Arun Narayanankutty, Juan M. Reséndiz-Hernández, Gloria Pérez-Rubio, Alejandra Ramírez-Venegas, Karol J. Nava-Quiroz, Nora E. Bautista-Félix, Gilberto Vargas-Alarcón, Manuel D. J. Castillejos-López, Andrés Hernández

**Affiliations:** ^1^HLA Laboratory, Instituto Nacional de Enfermedades Respiratorias Ismael Cosío Villegas, 14080 Ciudad de México, Mexico; ^2^Tobacco Smoking and COPD Research Department, Instituto Nacional de Enfermedades Respiratorias Ismael Cosío Villegas, 14080 Ciudad de México, Mexico; ^3^Molecular Biology Department, Instituto Nacional de Cardiología Ignacio Chávez, 14080 Ciudad de México, Mexico; ^4^Hospital Epidemiological Surveillance Unit, Instituto Nacional de Enfermedades Respiratorias Ismael Cosío Villegas, 14080 Ciudad de México, Mexico

## Abstract

**Background:**

The influenza A H1N1/09 pandemic infected a small number of exposed individuals, which suggests the involvement of genetic factors. There are scarce data available on classical HLA class I association with the influenza A H1N1/09 pandemic.

**Methods:**

We analyzed the frequency of classical HLA class I alleles and haplotypes in A H1N1/09 influenza in a case-control study including 138 influenza patients (INF-P) and 225 asymptomatic healthy contacts (INF-C) simultaneously recruited. HLA class I typing was performed by high-resolution sequence-based typing method.

**Results:**

Our analysis revealed higher frequency of C∗07:02:01, B∗39:06:02, C∗03:02:01, B∗44:03:01, B∗51:01:05, and B∗73:01 (*p* < 0.05; OR = 1.84–9.98) and of two haplotypes—A∗68:01:02-C∗07:02:01 (*p* = 1.05*E* − 05; OR = 23.99) and B∗35:01:01-C∗07:02.01 (*p* = 4.15*E* − 04, OR = 2.15)—in A H1N1/09 influenza subjects. A∗68:01:01 was exclusively present only in the INF-P group (5/138). A decrease in the frequency of C∗03:03:01, A∗11:01:01, B∗39:01:01, A∗24:02:01, C∗03:04:01, B∗51:01:01, and C∗07:01:01 (*p* < 0.05; OR = 0.12–0.52) and of haplotypes A∗02:01:01-B∗35:01:01-C∗04:01:01, A∗24:02:01-B∗35:01:01, B∗39:01:01-C∗07:02:01, and B∗40:02:01-C∗03:04:01 (*p* < 0.05; OR = 0.08–0.22) were observed in INF-P group.

**Conclusion:**

Selective classical HLA class I allele and haplotype combinations predispose individuals towards susceptibility or protection against the influenza A H1N1/09 pandemic. This work has significant implications for accessing population transmission risk for A H1N1/09 or a similar strain breakout in the future.

## 1. Introduction

Influenza A H1N1/09 pandemic, with its origin in Mexico, is the first human flu in 40 years to have caused such transmissibility and infectivity on a global scale [1, 2]. However, the mortality rates were merely marginal compared to those of previous global outbreaks. For example, the first well-known Spanish flu outbreak at 1918-1919 killed at least 40 million people globally, more than the total mortality rate attributed to World War I [3, 4]. The infection rate in various ethnic populations seems to be disproportionate along with the inability to identify any country-based transmission risk factors [2]. It has been previously shown that community and/or individual host factors can account for differential mortality rate [5]. Approximately 2/3 of global hospitalized subjects and 40% of fatal cases did not have any comorbidities or risk factors associated with influenza [6]. This may point to an underlying complex genetic component interplay associated with disease susceptibility. Several studies pointed towards the existence of a genetic component [7–9]; in this paper we analyze classical HLA class I region.

The differential specificity, selectivity, and diversity of human leukocyte antigen (HLA) directly reflect a delicate balance between interplay of molecular defense mechanisms against foreign antigens and autoimmunity acquired in the course of human evolution and migration. It is evident from various studies that selective combinations of HLA class I alleles exert quantitatively different effects on disease progression of infective or autoimmune origin [10–12]. HLA class I is involved in both innate (NK cells) and cell-mediated (CD8+ cells) immune response, significantly contributing towards viral clearance and decrease in the severity of influenza infection [4, 13].

Recent studies indicate that the NK cell frequency negatively correlates with the severity of influenza A H1N1/09 infection [13, 14]. NK cells attack virus-infected cells using a broad range of stimulatory and inhibitory receptor-ligand interactions. Quantitative interactions between selective HLA class I ligands and KIR, a major receptor of NK cell, variably influence the NK cell response towards viral-infected cells. Certain KIR3DL1/S1 allotypes, 3DL1/S1 and 2DL1 ligand-negative pairs, and 2DL2/L3 ligand-positive pairs are shown to be differentially enriched in severe cases of A H1N1/09 infection [15]. However, the less-studied KIRs are more polymorphic and diverse than are their classical HLA class I ligand counterparts. CD8+ effector T cell frequencies are shown to be normal regardless of patient disease status and severity [13]. However, it can be argued that there exist entirely different clones of CD8+ cells that correlate with H1N1 infection severity, and selective HLA class I alleles and combinations influence their generation/maintenance. This argument can be substantiated by the study showing that cytotoxic T lymphocytes established by seasonal influenza that cross-react against H1N1/09 are disproportionately distributed in a population [16]. Also, there are numerous reports of cross-reactive CTLs to heterosubtypic influenza A strains [17–20].

Currently, there are no studies in the Mexican population using direct sequencing on the relationship between HLA class I allele frequency and susceptibility to influenza virus A H1N1/09 infection. This is the first report that shows HLA class I allele association with susceptibility to influenza A H1N1/09 in Mexican mestizo population. Identification of alleles and haplotype associations with specific strains of influenza may help us to predetermine transmission rate of pandemic A H1N1/09 in various populations based on their HLA class I allele frequency.

## 2. Materials and Methods

### 2.1. Ethics Statement

This study was approved by the Institutional Committee for Science and Bioethics of the Instituto Nacional de Enfermedades Respiratorias (INER) Ismael Cosío Villegas (code B05-10). The study protocol was explained to all participants, and signed informed consent was duly obtained.

All the information was collected complying with Official Mexican Standards (Mexicana NOM-168-SSA1-1998); topics covered included age, gender, tobacco smoking, body mass index (BMI; patients with BMI > 30 kg/m^2^), and disease morbidity (pulmonary, hepatic, renal, cardiac, and neurological diseases, diabetes mellitus, hypertension, and cancer). The symptoms evaluated were fever, cough, rhinorrhea, dyspnea, nasal congestion, thoracic pain, headache, diarrhea, and vomiting. The start of antiviral therapy was evaluated in relation to the days with previous symptomatology. The laboratory parameters included were leukocyte titer, lactate dehydrogenase (LDH), creatine phosphokinase (CPK), blood urea nitrogen (BUN), and arterial gases (with PaO_2_ < 60 mmHg defined as severe disease). Pneumonia was verified by radiological findings. All patients admitted to the intensive care unit (ICU) and those put on assisted mechanical ventilation (AMV) were identified.

### 2.2. Study Subjects

**Figure 1:**
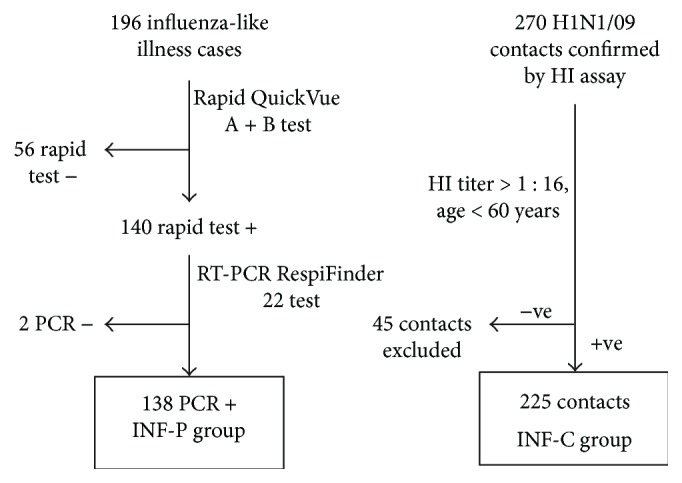
Selection criteria for both influenza A H1N1/09 patient (INF-P) and contact (INF-C) groups. INF-P: influenza patients; INF-C: influenza contacts; HI: hemagglutination inhibition; RT-PCR: RT-PCR: real-time polymerase chain reaction, also called quantitative PCR (qPCR).

A total of 196 subjects showing influenza-like illness were enrolled in the study during the first and second waves of influenza A H1N1/09 infection in Mexico (between April and December 2009). All the subjects included in the study were Mexican mestizos by ethnicity with at least 2 generations born and brought up in Mexico. The 196 study subjects were classified into two categories: 138 H1N1/09 positive (influenza A H1N1/09 positive subjects [INF-P]) and 58 H1N1/09 negative subjects; only the INF-P group was enrolled in the study. Another group of 225 subjects (asymptomatic healthy contacts [INF-C]) was also enrolled in parallel during the same period (Figure 1). Anti-influenza A H1N1 antibody titers were determined to ensure that those in the INF-C group had been exposed to the virus.

### 2.3. Immunological and Viral Measurements

#### 2.3.1. INF-P Group (*n* = 138)

Nasopharyngeal swabs were taken from the subjects and eluted in 800 *μ*L saline by vigorously rotating the swab in the diluent. Each sample was analyzed using rapid QuickVue Influenza A + B test (Quidel, CA, USA) following the recommendations for collection and testing from US Centers for Disease Control and Prevention (CDC) and World Health Organization (WHO). Positive cases for influenza A H1N1 confirmed by QuickVue were further validated using an RT-PCR-based RespiFinder 22 test (Maastricht, Netherlands) [21].

#### 2.3.2. INF-C Group (*n* = 225)

Although not biologically related to any patients in the INF-P group, they were in personal contact with one or more of A H1N1-infected patients. However, they never showed any symptoms of flu or took any medication for it. Hemagglutination inhibition (HI) assay was performed using peripheral blood samples for each subject as previously described [22]. All subjects included in this group had an HI titer value greater than 1 : 16 dilutions, indicating direct contact with the virus. All INF-C subjects were under 60 years of age to avoid any cases of cross-reacting antibodies prevalent in older age groups from H1N1/1918-1919 infection [23]. As this cohort was not progressively monitored for the dynamics of anti-A H1N1/09 antibody titer over time, we will not show the results.

### 2.4. Anti-A H1N1/09 Antibody Titers

Serially diluted aliquots of serum samples (25 *μ*L) in PBS were mixed with 25 *μ*L aliquots of the A H1N1/09 virus strain (corresponding to four hemagglutination units). The serum/virus dilutions were incubated for 30 min at room temperature. 50 *μ*L of 0.5% chicken erythrocytes was added, and after 30 min, the HI activity was evaluated. The serum HI antibody titer was established as the reciprocal of the last serum dilution with no hemagglutination activity. Those individuals with titers greater than 1 : 16 were considered positive for A/H1N1 infection/exposure.

### 2.5. Population HLA Frequency Data

HLA frequency data for Mexican mestizos were obtained from the http://www.allelefrequencies.net website [24]. Average HLA frequencies were computed using a weighted average of all reported studies, based on study sample size.

### 2.6. DNA Extraction and HLA Class I Typing

High-molecular-weight genomic DNA was extracted from peripheral blood using BDtract genomic DNA isolation kit (Maxim Biotech, San Francisco, CA, USA). All DNA samples were stored indefinitely at −20°C in Tris-EDTA buffer (10 mM Tris-HCl, 2 mM EDTA, pH 8.0) until further use.

### 2.7. HLA Class I Typing

Genomic DNA (50 ng/*μ*L) was amplified using long-range PCR (LR-PCR) for each HLA-A, HLA-B, and HLA-C loci using AlleleSEQR core reagent pack according to the manufacturer's protocol. HLA-A, HLA-B, and HLA-C alleles were determined by a sequence-based typing (SBT) method, using the AlleleSEQR HLA-A, AlleleSEQR HLA-B, and AlleleSEQR HLA-C plus sequencing kits, respectively (Atria Genetics Inc., San Francisco, CA, USA). The cycling conditions used were 95°C 10 min, 1 cycle; 96°C 20 sec, 60°C 30 sec, and 72°C 3 min for 36 cycles; and 4°C until the next step. The 2 kb amplicons were verified by 1% agarose gel electrophoresis. The product obtained was treated with ExoSAP-IT, a mixture of an exonuclease I/alkaline phosphatase, in order to remove unincorporated primers and dNTPs, using one cycle of 37°C 30 min, 80°C 15 min, and 4°C until the next step. Exons 2, 3, and 4 were amplified with specific degenerate primers for HLA-A, HLA-B, and HLA-C loci using the following cycling conditions: twenty-five cycles at 96°C 20 sec, 50°C 30 sec, and 60°C 2 min and 4°C until the next step. Samples were prepared for loading by adding 15 *μ*L of formamide and denaturing at 95°C for 2 min.

Sequencing was performed on a ABI3130 Genetic Analyzer (Applied Biosystems, Foster City, CA, USA) using mobile file v2.mob DT3130POP6{BD}, with an analysis module RapidSeq36_POP6_1, 1–1.5 kV injecting 5–10 seconds, collecting data for 1800 seconds and reviewing it with the Sequencing Analysis software v5.4. The interpretation and assignment of HLA alleles were performed using Assign v3.5 software (Conexio Genomics), with reference sequences updated to April 2012 from the IMGT database.

### 2.8. Statistical Analysis

Allele and haplotype frequencies of HLA class I were enumerated by direct counting in both INF-P and INF-C study groups. The observed and expected HLA class I alleles (each *locus*) were tested for Hardy-Weinberg equilibrium using a conventional Fisher's exact test. Differential allele and haplotype frequency associations between different HLA class I allele variants were assessed using Fisher's exact two-tailed test with a significance cutoff level of *p* < 0.05. Bonferroni correction was carried out by only taking into account alleles with allele frequency greater than 5% to compensate for overcorrection. Odd ratios and 95% confidence intervals were calculated using Epi Info 7.1.3.0 software. Allele frequency graphs were plotted using SigmaPlot 11. Finally, the haplotypes (2-locus and 3-locus) were constructed employing Arlequin v3.1 software [25] using the maximum likelihood method, with an iterative EM algorithm.

## 3. Results

We analyzed clinical data from 138 influenza patients and 225 healthy contacts, as described in Table 1. No statistically significant difference was observed for age between the study groups (43.26 versus 42.21, *p* = 0.667). The male to female ratios of INF-P and INF-C were 1.3 : 1 and 1 : 1.14, respectively (*p* = 0.069). As described previously [6], a statistically significant difference was observed in the variables of weight (83.41 versus 71.0, *p* < 0.01), height (1.64 versus 1.59, *p* < 0.01), BMI (30.88 versus 27.85, *p* = 0.01), and active smoking status (45.65% versus 30.61%, *p* = 0.02). Comorbidities and symptomatology characteristics are described in Table 1. Eighty-nine patients (64.49%) had pneumonia, and 71 (51.44%) were admitted to the ICU, whereas thirty-one patients positive for influenza A H1N1/09 virus died. None of the alleles/haplotypes were directly associated with mortality or greater severity.

**Figure 2:**
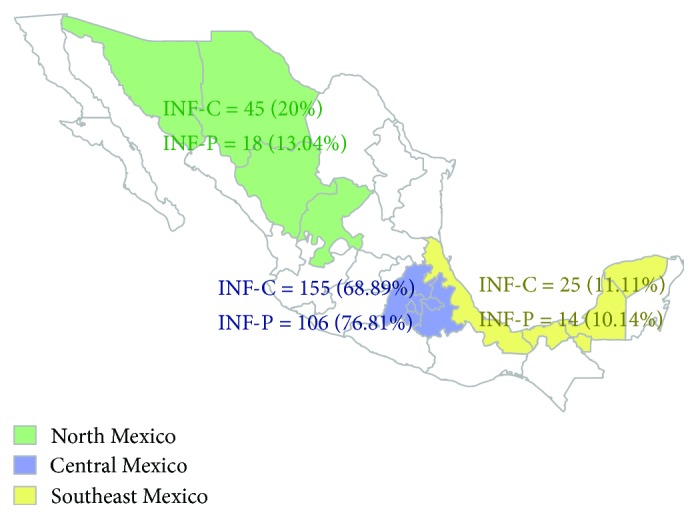
Depiction of influenza A H1N1/09 patient (INF-P) and contact (INF-C) organized to three strata based on their region of origin. INF-P: influenza patients; INF-C: influenza contacts.

In order to avoid population stratification effects, cases and contacts were compared with respect to their region of origin, grouping them as coming from central (states of Mexico, Hidalgo, Puebla, Morelos, Tlaxcala, and Mexico City), north (states of Chihuahua, Sonora, Durango, and Zacatecas), and southeast (states of Veracruz, Tabasco, Campeche, and Yucatan) regions (Figure 2). 76.81% of the INF-P and 68.9% of INF-C were from the central area of the country contributing to majority of subjects in both the study groups (*p* = 0.104). The northern states contributed 13.04% of INF-P and 20% of INF-C (*p* = 0.131). Finally, 10.14% of INF-P subjects were from the southeastern states of Mexico, compared to 11.11% INF-C (*p* = 0.610) (Figure 2). No statistically significant differences were found in the population stratification based on region of origin between cases and contacts.

### 3.1. Differential Frequency Distribution of HLA Class I Alleles

The HLA allele pairs of each locus from both groups satisfied the Hardy-Weinberg equilibrium (*p* > 0.05), accounting for common alleles producing common genotypes [i.e., both heterozygous (e.g., B∗35:01:01 and B∗39:01:01) and homozygous (e.g., A∗02:01:01) pairs].

Our analysis identified 31 HLA-A alleles in influenza patients (INF-P) and 38 in asymptomatic healthy contacts (INF-C) out of which 7 of the alleles in the INF-P group showed an allele frequency (AF) higher than 6%; the most common were A∗02:01:01 (24.6%), A∗68:01:02 (11.6%), A∗24:02:01 (7.9%), A∗03:01:01 (7.6%), A∗31:01:02 (7.2%), and A∗01:01:01 (6.5%). Interestingly, A∗68:01:01 allele was present exclusively in the INF-P (AF = 1.8%). Furthermore, we observed a lower frequency of the allele A∗24:02:01 (7.9% versus 18.6% *p*_corr_ = 1.06*E* − 03) in the INF-P compared to INF-C, respectively, suggesting a possible protective role against the A H1N1/09 virus infection (OR = 0.38). A lower frequency of A∗11:01:01 (0.4% versus 2.8%, *p* = 1.63*E* − 02) was also observed in INF-C but did not reach statistical significance after Bonferroni correction (Table 2).

HLA-B *locus* typing identified 42 alleles in INF-P and 57 alleles in INF-C. The most frequent were B∗35:01:01, B∗15:01:01, and B∗07:02:01 in both study groups; in addition, B∗39:01:01 and B∗51:01:01 were common in the INF-C group. Increased frequency of B∗51:01:05 (*p*_corr_ = 3.04*E* − 02, OR = 6.18) was detected in the INF-P group compared with INF-C. B∗39:06:02 (*p* = 2.12*E* − 02), B∗44:03:01 (*p* = 3.55*E* − 03), and B∗73:01 (*p* = 8.99*E* − 03) were also increased in the INF-P group but failed to reach significance after Bonferroni correction (OR = 2.53–9.98). In contrast, a decreased frequency of the alleles B∗51:01:01 (*p* = 2.25*E* − 02, OR = 0.43) and B∗39:01:01 (*p*_corr_ = 2.09*E* − 02, OR = 0.26) was observed in INF-P compared to INF-C (Table 3).

HLA-C *locus* typing identified 26 alleles in INF-P and 21 in INF-C. The molecular subtypes C∗07:02:01, C∗04:01:01, C∗01:02:01, and C∗08:01:01 represent more than 55% of the specificities detected in INF-P patients. We observed an increased frequency of C∗07:02:01 (*p*_corr_ = 3.68*E* − 02) and C∗02:02:02 (*p*_corr_ = 2.70*E* − 02) alleles in the INF-P group compared with INF-C. A decrease in the frequency of the subtype C∗03:04:01 (*p*_corr_ = 4.30*E* − 02) was detected in the INF-P group. C∗07:01:01 (*p* = 3.67*E* − 02) and C∗03:03:01 (*p* = 8.34*E* − 03) were also decreased but failed to reach Bonferroni significance; something similar happened with C∗03:02:01 and C∗08:01:02, which are statistically increased in the patients group (*p* = 1.04*E* − 02 and 4.10*E* − 03, resp.) (Table 4).

### 3.2. Differential Frequency Distribution of Haplotypes (*Loci* A-B-C, A-B, A-C, and B-C)

We identified 211 HLA-A-B-C haplotypes in the INF-P and 276 combinations in the INF-C, respectively. In the INF-P group, only 10 combinations showed a haplotype frequency (HF) > 1%; the most common were A∗02:01:01-B∗35:01:01-C∗07:02:01 (2.54%), A∗68:01:02-B∗48:01:01-C∗08:01:01 (1.81%), A∗01:01:01-B∗08:01:01-C∗07:02:01 (1.45%), A∗03:01:01-B∗35:01:01-C∗04:01:01 (1.45%), A∗24:02:01-B∗15:01:01-C∗01:02:01 (1.45%), A∗02:01:01-B∗15:01:01-C∗07:02:01 (1.45%), and A∗32:01:01-B∗35:01:01-C∗04:01:01 (1.45%). Similarly, 16 haplotypes had an HF > 1% in the INF-C group; the most frequent were A∗02:01:01-B∗35:01:01-C∗04:01:01 (2.57%), A∗24:02:01-B∗35:01:01-C∗04:01:01 (2.64%), A∗02:01:01-B∗39:01:01-C∗07:02:01 (2.38%), A∗24:02:01-B∗35:01:01-C∗03:04:01 (1.99%), and A∗03:01:01-B∗07:02:01-C∗07:02:01 (1.33%).

We compared the HF of HLA-A-B-C *loci* observed in INF-P with that observed in INF-C (Table 5). Our analysis revealed that the combination A∗02:01:01-B∗35:01:01-C∗04:01:01 was diminished significantly in INF-P (*p* = 0.021, OR = 0.13, CI = 0.01–0.91); however, after Bonferroni correction (HF > 1%), *p* value was not statistically significant (*p* = 0.219). Interestingly, 2 haplotypes were present at high frequency (~2.0%) in the INF-C group and completely absent among the INF-P; these include A∗24:02:01-B∗35:01:01-C∗03:04:01 and A∗24:02:01-B∗35:01:01-C∗04:01:01 (see Tables SIX–SXII, Supplementary Materials); it is not possible to make statistical comparisons between groups as the haplotype frequencies readily decline due to extensive sample size dilution. However, the last two haplotypes carry A∗24:02:01 and C∗03:04:01 alleles, both previously associated with protection in the individual *locus* analysis.

In a separate analysis of A-B, A-C, and B-C haplotypes, few combinations had frequencies greater than 2%. We observed that the haplotype frequencies of A∗24:02:01-B∗35:01:01, B∗39:01:01-C∗07:02:01, and B∗40:02:01-C∗03:04:01 were diminished in the INF-P compared to INF-C (*p* < 0.05 uncorrected). Two highly significant haplotypes—A∗68:01:02-C∗07:02:01 (pC = 2.53*E* − 04, OR = 23.99, CI = 3.14–183.51) and B∗35:01:01-C∗07:02:01 (pC = 6.64*E* − 03, OR = 16.88, CI = 2.14–132.60)—were observed in the INF-P, suggesting that these haplotypes are important genetic susceptibility factors predisposing them to symptomatic influenza A H1N1/09 viral infection. Three haplotypes (HF > 2.0%)—A∗68:01:02-B∗35:01:01, A∗68:01:02-B∗51:01:05, and B∗08:01:01-C∗07:02:01—are increased in the INF-P group and two among contacts, A∗24:02:01-C ∗ 03:04:01 and B∗51:01:01-C∗15:02:01. However, it is not possible to make statistical comparisons between groups. For a complete list of haplotypes, check the Supplementary Materials.

### 3.3. General Mexican Mestizo Population HLA Class I Allele Frequency Compared with INF-P and INF-C

**Figure 3:**
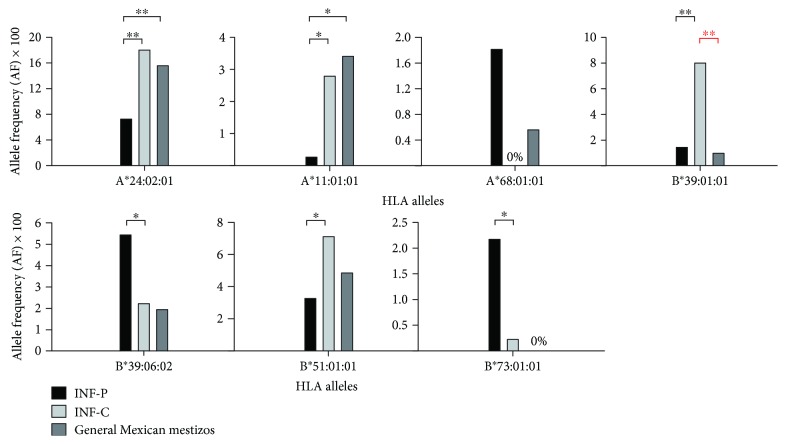
Allele frequency distribution in general Mexican mestizo population compared to influenza A H1N1/09 patient (INF-P) and contact (INF-C). Statistically significant alleles in the comparison of INF-P to INF-C with available 6-digit allele frequency data for Mexican mestizo population are included. INF-P: influenza patients; INF-C: influenza contacts; HLA: human leukocyte antigen. ^∗^ *p* value < 0.05; ^∗∗^ *p* value < 0.01. All *p* values were obtained by Fisher exact two-tailed test.

Statistically significant alleles from the INF-P versus INF-C group were compared with population allele frequency for Mexican mestizos. However, B∗51:01:05, B∗41:03:01, and none of the HLA-C alleles were compared as there were no six-digit resolution data available. B∗39:01:01 showed statistically significant variation from the INF-C group (*p* < 0.05), indicating allele enrichment different from that of the general population (Figure 3). The sample size of HLA class I reported studies was significantly lower compared to sample size of our study. A stringent Fisher's exact two-tailed test was used to calculate the *p* values. This may have under-/overrepresented the actual allele frequencies.

## 4. Discussion

In this study, we identified several classical HLA class I alleles and haplotypes that may confer susceptibility or protection against A H1N1/09 infection. We analyzed distribution of classical HLA class I (A, B, and C) *loci* in 138 patients with influenza A H1N1/09 infection and 225 asymptomatic contacts. As expected, we found enrichment of specific alleles and its combinations in the INF-P group.

According to the primary reports of the A H1N1/09 outbreak in Mexico, subjects aged between 10 and 19 years were at increased risk of infection [26]. However, our study subjects do not show this trend. INER has one of the most important respiratory critical care hospitals in Mexico. As a result, severe/complicated cases are referred more to this hospital, hence contributing towards offsets from the previous reports. Increased BMI and smoking were more frequent in influenza patients, which agrees with previous studies [6].

Our analysis revealed lower frequency of A∗24:02:01 in patients compared to asymptomatic contacts, suggesting a possible protective effect. This result is in disaccord with the study showing A∗24 (A24 family) positively correlating with severe A H1N1 infection and mortality [27]. However, that result was obtained employing database analysis of conserved proteomic regions-based experimental predictions of HLA binding affinity, showing that HLA alleles preferentially target conserved regions of viral proteins (HLA targeting efficiency) [28]. The study did not consider HLA-restricted NK cell-mediated viral clearance, which may have introduced a certain degree of bias. The HLA-A∗24 subtype is a potential ligand for KIR3DL1, rendering an inhibitory NK signal [29]. This may reduce the overreactivity of immune system to influenza A H1N1/09 virus, which is known to contribute to the significant pathobiology of the disease [15, 30]. Also, A∗24:02 is in linkage disequilibrium with B∗39:01 [31], which possesses high targeting efficiency scores. A∗24 comprises a large portion of the world population; this allele family is more common in certain indigenous group and constitutes more than half of the global population, especially in Asian countries and several Native American populations [32]. In Mexican mestizos, there exist heterogeneous A∗24 frequencies depending on the Amerindian contribution and the region studied [31]. In this study, only 8.33% of the patients had any allele of A∗24 serotype whereas the contact subjects reach 18% (A∗24:02:01). The general allele frequency of A∗24:02:01 in Mexican mestizos is 15% [33] (Figure 3).

Our HLA dataset contains six A∗68 alleles, out of which two belong to different supertypes: A∗68:01 to the A3 supertype and A∗68:02 to the A2 supertype. No association was found with A∗68:02:01 to A H1N1/09 infection, while A∗68:01:01, despite its low frequency (1.8%), was only present in patients. This is in concordance with the results of the HLA targeting efficiency study showing the A∗68:01 subtypes positively correlating with A H1N1/09 mortality rates but not A∗68:02 [27, 34]. Also, we did not find any relationship between A∗02:01:01 and for that matter any A∗02 subtypes against A H1N1 infection, which is considered to be a general protection factor against influenza [35].

Higher frequencies of B∗39:06:02 (statistically insignificant after Bonferroni correction) and B∗51:01:05 were observed in subjects suffering from influenza A H1N1. In spite of high targeting efficiency scores, B∗39:06:02 (Bw6) allele frequency was higher in A H1N1 patients compared to asymptomatic contacts. We suspect that this may be due to selective pressure exerted by certain KIR-HLA combinations favoring underreactivity of NK cells. Interestingly, the allele B∗39:06 has been observed in several Amerindians populations with a frequency of around 5% [31] and possesses high targeting efficiency score [27]. In contrast, B∗39:01:01, another Bw6 supertype, showed a protective effect; this observation may be due to strong linkage disequilibrium with A∗24:02 existing in Amerindian populations. A higher frequency of B∗51:01:05 was also found in INF-P patients. Another allele, HLA-B∗51:01:01 (not significant after Bonferroni correction), an ancestral allele of Amerindian origin [31], had low frequency in A H1N1/09 patients, pointing towards a possible protective role. In contrast with the HLA-A alleles, the HLA-B alleles bind more efficiently with the conserved regions of the A H1N1 viral proteins. Molecular subtypes of HLA-B∗39 (B∗39:01 and B∗39:06), common in Amerindian populations, are apparently associated with severe forms of the disease [27], which may be a result of these alleles in linkage disequilibrium with another region within or close to HLA *locus*.

High frequency of C∗03:02:01 was observed in A H1N1/09 patients, despite its very low frequency in the Mexican population [31]; however, this is not statistically significant after Bonferroni correction. On the other hand, C∗03:03:01, C∗03:04:01, and C∗07:01:01 showed low frequency in the INF-P group. HLA-C, compared to HLA-A or HLA-B, is less polymorphic and presents more restricted repertoire of peptides and low cell surface expression [31, 36]. Proportionally higher frequency of KIR2DL1 C2− C1+ and KIR2DL3 C1+ was reported in ICU A H1N1/09 patients (indigenous and nonindigenous), relative to healthy controls [15].

Relatively low frequency of the A∗02:01:01-B∗35:01:01-C∗04:01:01 haplotype was observed in the A H1N1/09 patients. This specific HLA haplotype represents more than 2.5% in the control group and has been found only in Hispanic and Mexican populations with the resolution employed in this study. We observed an increase in the frequency of A∗68:01:02-C∗07:02:01 haplotype in the patients. This finding is relevant because linkage disequilibrium is very high in the region rendering potential haplotypes and can potentially amplify the risk to disease. Considering the resolution employed in the study, it is difficult to compare functional and population-based allele/haplotype frequencies.

Our study was subjected to the following limitations; first, we did not consider risk factors associated with influenza other than obesity, smoking status, and pregnancy. Various diseases such as asthma, diabetics, immunodeficiency, and pneumococcal pneumonia are all risk factors for A H1N1/09 influenza [6, 37]. Second, the sample size of each group and the general allele frequency was low, which may have affected the power of statistical analysis to a certain degree. Third, the antibody titer observed in the contacts group may have contributions from seasonal influenza strains. The exact degree of contribution from cross-reacting antibodies from seasonal strains is largely unknown [38]. However, we expect that the contribution is minor and could not reach a threshold of 1 : 16 dilutions. Surveillance of circulating A H1N1/pdm09 viruses has revealed some genetic variations in the viral surface glycoproteins, hemagglutinin, and neuraminidase. However, until recently, the antigenicity of the circulating A H1N1/pdm09 viruses was similar to the vaccine strain (A/California/7/2009) in assays with panels of antiserum obtained from infected ferrets [39]. Despite these limitations, this is the first report showing HLA class I alleles and haplotypes associated with susceptibility to influenza A H1N1/09 in the Mexican population.

In summary, B∗51:01:05, C∗07:02:01, and C∗02:02:02 were all increased among A H1N1/09-infected patients (INF-P), while A∗24:02:01, B∗51:01:01, B∗39:01:01, and C∗03:04:01 were elevated in asymptomatic healthy contacts. B∗39:01:01 showed a statistically significant variation from the INF-C group compared to general Mexican mestizos. Haplotype analysis revealed enrichment of certain allele combinations out of which A∗68:01:02-C∗07:02:01 and B∗35:01:01-C∗07:02:01 were found to be important haplotypes susceptible to A H1N1/09 infectivity.

Our study validates that selective classical HLA class I alleles and haplotypes are favored for both susceptibility and protection against A H1N1/09 pandemic. From our results, we speculate that the selective pressure on HLA class I enrichment may be a result of specific KIR-HLA combinations and HLA targeting efficiency along with other factors. Further studies may be needed to validate the result and elucidate the role of HLA class I alleles in A H1N1/09 influenza.

## Figures and Tables

**Table 1:** Characteristics of influenza A H1N1/09 patients (INF-P) and contacts (INF-C) recruited during April to December 2009.

	INF-P (*n* = 138)	INF-C (*n* = 225)	*p*
Age	43.26 ± 13.86	42.21 ± 12.03	
Gender [male (%)/female (%)]	78 (56.52)/60 (43.48)	105 (46.67)/120 (53.33)	
Weight (kg)	83.41 ± 20.45	71.00 ± 14.10	<0.01
Height (m)	1.64 ± 0.10	1.59 ± 0.09	<0.01
BMI	30.88 ± 6.92	27.85 ± 4.86	0.01
Smoking (Y/N, %)	45.65/54.35	30.61/69.39	0.02
Comorbidities (%)			
Neurological disease	25 (18.11)	33 (14.66)	
Asthma	2 (1.44)	6 (2.66)	
Cancer	1 (0.72)	0 (0.00)	
Hypertension	8 (5.79)	17 (7.55)	
Symptomatology (%)			
Fever (>38°C)	104 (75.36)	0 (0.00)	
Cough	72 (52.17)	0 (0.00)	
Nasal congestion	17 (12.31)	0 (0.00)	
Rhinorrhea	39 (28.26)	0 (0.00)	
Dyspnea	101 (73.18)	0 (0.00)	
Region of origin; *n* (%)			
Central Mexico	106 (76.81)	155 (68.89)	
North Mexico	18 (13.04)	45 (20.00)	
Southeast Mexico	14 (10.14)	25 (11.11)	

INF-P: influenza patients; INF-C: influenza contacts; *n*: number of subjects; BMI: body mass index. *p* value < 0.05 is considered significant.

**Table 2:** HLA-A allele frequencies in patients with influenza A/H1N1 infection (INF-P) and asymptomatic healthy contacts (INF-C).

HLA-A	INF-P *n* = 138			INF-C *n* = 225			*p*	pC	OR	95% CI
	*n*	AF	(%)	*n*	AF	(%)				
A∗02:01:01	68	0.2464	24.638	120	0.2667	26.667				
A∗68:01:02	32	0.1159	11.594	41	0.0911	9.111				
A∗24:02:01	22	0.0797	7.971	84	0.1867	18.667	7.56*E* − 05	1.06*E* − 03	0.3774	0.2298–0.6197
A∗03:01:01	21	0.0761	7.609	27	0.0600	6.000				
A∗31:01:02	20	0.0725	7.246	22	0.0489	4.889				
A∗01:01:01	18	0.0652	6.522	23	0.0511	5.111				
A∗02:06:01	17	0.0616	6.159	19	0.0422	4.222				
A∗29:02:01	10	0.0362	3.623	10	0.0222	2.222				
A∗26:01:01	8	0.0290	2.899	8	0.0178	1.778				
A∗32:01:01	8	0.0290	2.899	7	0.0156	1.556				
A∗30:02:01	7	0.0254	2.536	10	0.0222	2.222				
A∗23:01:01	6	0.0217	2.174	14	0.0311	3.111				
A∗26:01:02	5	0.0181	1.812	4	0.0089	0.889				
A∗68:01:01	5	0.0181	1.812	0			4.17*E* − 03	5.84*E* − 02	Und	Und-Und
A∗01:03	3	0.0109	1.087	1	0.0022	0.222				
A∗11:24:02	3	0.0109	1.087	0						
A∗33:01:02	3	0.0109	1.087	11	0.0244	2.444				
A∗66:02	3	0.0109	1.087	0						
A∗68:02:01	3	0.0109	1.087	1	0.0022	0.222				
A∗25:01:01	2	0.0072	0.725	3	0.0067	0.667				
A∗33:01:01	2	0.0072	0.725	0						
A∗02:04	1	0.0036	0.362	0						
A∗02:05:01	1	0.0036	0.362	0						
A∗11:01:01	1	0.0036	0.362	13	0.0289	2.889	1.63*E* − 02	2.28*E* − 01	0.1222	0.0159–0.9397
A∗23:01:02	1	0.0036	0.362	0						
A∗23:02	1	0.0036	0.362	0						
A∗24:02:06	1	0.0036	0.362	0						
A∗30:01:01	1	0.0036	0.362	1	0.0022	0.222				
A∗32:02:01	1	0.0036	0.362	1	0.0022	0.222				
A∗68:03:01	1	0.0036	0.362	1	0.0022	0.222				
A∗74:02:01	1	0.0036	0.362	0						
A∗01:01:02	0			2	0.0044	0.444				
A∗02:01:02	0			1	0.0022	0.222				
A∗02:02:01	0			3	0.0067	0.667				
A∗02:03:01	0			1	0.0022	0.222				
A∗11:01:02	0			1	0.0022	0.222				
A∗11:04	0			1	0.0022	0.222				
A∗26:02:01	0			1	0.0022	0.222				
A∗31:02	0			4	0.0089	0.889				
A∗31:14N	0			1	0.0022	0.222				
A∗36:01	0			4	0.0089	0.889				
A∗66:01:01	0			3	0.0067	0.667				
A∗68:01:03	0			1	0.002	0.222				
A∗68:03:02	0			1	0.002	0.222				
A∗68:05	0			1	0.002	0.222				
A∗69:01	0			1	0.002	0.222				
A∗74:01	0			2	0.004	0.444				
A∗80:01	0			1	0.002	0.222				

INF-P: influenza patients; INF-C: influenza contacts; HLA: human leukocyte antigen; AF: allele frequency; *n*: number of subjects; OR: odds ratio; CI: confidence interval; pC: *p* value after Bonferroni correction; Und: undefined; only showing *p* value < 0.05, which is considered significant.

**Table 3:** HLA-B allele frequencies in patients with influenza A/H1N1 infection (INF-P) and asymptomatic healthy contacts (INF-C).

HLA-B	INF-P *n* = 138			INF-C *n* = 225			*p*	pC	OR	95% CI
	*n*	AF	(%)	*n*	AF	(%)				
B∗35:01:01	37	0.1341	13.406	64	0.1422	14.222				
B∗15:01:01	26	0.0942	9.420	33	0.0733	7.333				
B∗07:02:01	17	0.0616	6.159	24	0.0533	5.333				
B∗48:01:01	17	0.0616	6.159	20	0.0444	4.444				
B∗39:06:02	15	0.0543	5.435	10	0.0222	2.222	2.12*E* − 02	4.03*E* − 01	2.5287	(1.1197–5.7111)
B∗40:02:01	14	0.0507	5.073	26	0.0578	5.778				
B∗51:01:05	11	0.0399	3.986	3	0.0067	0.667	1.60*E* − 03	3.04*E* − 02	6.1849	(1.7100–22.3706)
B∗44:03:01	10	0.0362	3.623	3	0.0067	0.667	3.55*E* − 03	6.74*E* − 02	5.6015	(1.5279–20.5356)
B∗52:01:02	10	0.0362	3.623	15	0.0333	3.333				
B∗39:05:01	9	0.0326	3.261	8	0.0178	1.778				
B∗51:01:01	9	0.0326	3.261	33	0.0733	7.333	2.25*E* − 02	4.28*E* − 01	0.4259	(0.2006–0.9043)
B∗08:01:01	8	0.0290	2.899	10	0.0222	2.222				
B∗14:02:01	7	0.0254	2.536	11	0.0244	2.444				
B∗39:01:01	6	0.0217	2.174	36	0.0800	8.000	1.10*E* − 03	2.09*E* − 02	0.2556	(0.1062–0.6147)
B∗58:01:01	6	0.0217	2.174	3	0.0067	0.667				
B∗73:01	6	0.0217	2.174	1	0.0022	0.222	8.99*E* − 03	1.71*E* − 01	9.9778	(1.1948–83.3273)
B∗13:02:01	5	0.0181	1.812	4	0.0089	0.889				
B∗18:01:01	5	0.0181	1.812	9	0.0200	2.000				
B∗35:17	5	0.0181	1.812	4	0.0089	0.889				
B∗38:01:01	4	0.0145	1.449	6	0.0133	1.333				
B∗40:05	4	0.0145	1.449	1	0.0022	0.222				
B∗44:02:01	4	0.0145	1.449	16	0.0356	3.556				
B∗49:01:01	4	0.0145	1.449	7	0.0156	1.556				
B∗14:06:02	3	0.0109	1.087							
B∗15:03:01	3	0.0109	1.087	5	0.0111	1.111				
B∗35:02:01	3	0.0109	1.087	6	0.0133	1.333				
B∗39:06:01	3	0.0109	1.087	6	0.0133	1.333				
B∗40:01:01	3	0.0109	1.087	7	0.0156	1.556				
B∗14:01:01	2	0.0072	0.725	4	0.0089	0.889				
B∗27:03	2	0.0072	0.725	6	0.0133	1.333				
B∗27:05:02	2	0.0072	0.725	2	0.0044	0.444				
B∗35:05:01	2	0.0072	0.725	0						
B∗35:12:01	2	0.0072	0.725	9	0.0200	2.000				
B∗39:02:02	2	0.0072	0.725	3	0.0067	0.667				
B∗41:01:01	2	0.0072	0.725	4	0.0089	0.889				
B∗53:01:01	2	0.0072	0.725	2	0.0044	0.444				
B∗15:02:01	1	0.0036	0.362	4	0.0089	0.889				
B∗15:16:01	1	0.0036	0.362	0						
B∗35:01:02	1	0.0036	0.362	0						
B∗45:01:01	1	0.0036	0.362	5	0.0111	1.111				
B∗57:01:01	1	0.0036	0.362	6	0.0133	1.333				
B∗78:02:02	1	0.0036	0.362	0						
B∗13:01:01	0			1	0.0022	0.222				
B∗15:05:01	0			1	0.0022	0.222				
B∗15:15	0			2	0.0044	0.444				
B∗35:01:04	0			1	0.0022	0.222				
B∗35:40N	0			1	0.0022	0.222				
B∗37:01:01	0			1	0.0022	0.222				
B∗38:01:02	0			6	0.0133	1.333				
B∗40:01:02	0			1	0.0022	0.222				
B∗40:01:04	0			3	0.0067	0.667				
B∗40:02:03	0			1	0.0022	0.222				
B∗40:11:01	0			1	0.0022	0.222				
B∗42:01:01	0			2	0.0044	0.444				
B∗44:18:01	0			1	0.0022	0.222				
B∗45:01	0			1	0.0022	0.222				
B∗46:02	0			1	0.0022	0.222				
B∗50:01:01	0			3	0.0067	0.667				
B∗51:02:01	0			1	0.0022	0.222				
B∗55:01:01	0			3	0.0067	0.667				
B∗55:02:01	0			2	0.0044	0.444				
B∗56:01:01	0			1	0.0022	0.222				

INF-P: influenza patients; INF-C: influenza contacts; HLA: human leukocyte antigen; AF: allele frequency; *n*: number of subjects; OR: odds ratio; CI: confidence interval; pC: *p* value after Bonferroni correction; only showing *p* value < 0.05, which is considered significant.

**Table 4:** HLA-C allele frequencies in patients with influenza A/H1N1 infection (INF-P) and asymptomatic healthy contacts (INF-C).

Allele	INF-P *n* = 138			INF-C *n* = 225			*p*	pC	OR	95% CI
	*n*	AF	(%)	*n*	AF	(%)				
C∗07:02:01	60	0.2174	21.739	59	0.1311	13.111	2.30*E* − 03	3.68*E* − 02	1.8409	1.2390–2.7351
C∗04:01:01	50	0.1812	18.116	75	0.1667	16.667				
C∗01:02:01	22	0.0797	7.971	31	0.0689	6.889				
C∗08:01:01	22	0.0797	7.971	26	0.0578	5.778				
C∗07:01:01	14	0.0507	5.073	42	0.0933	9.333	3.67*E* − 02	5.88*E* − 01	0.5191	0.2780–0.9692
C∗03:04:01	12	0.0435	4.348	48	0.1067	10.667	2.69*E* − 03	4.30*E* − 02	0.3807	0.1985–0.7302
C∗03:02:01	11	0.0399	3.986	5	0.0111	1.111	1.04*E* − 02	1.67*E* − 01	3.6943	1.2697–10.7492
C∗08:02:01	11	0.0399	3.986	15	0.0333	3.333				
C∗16:01:01	11	0.0399	3.986	17	0.0378	3.778				
C∗06:02:01	10	0.0362	3.623	14	0.0311	3.111				
C∗05:01:01	8	0.0290	2.899	18	0.0400	4.000				
C∗12:03:01	8	0.0290	2.899	22	0.0489	4.889				
C∗02:02:02	6	0.0217	2.174	0			1.69*E* − 03	2.70*E* − 02	Und	Und-Und
C∗08:01:02	5	0.0181	1.812	0			4.10*E* − 03	6.56*E* − 02	Und	Und-Und
C∗15:02:01	5	0.0181	1.812	21	0.0467	4.667				
C∗03:03:01	4	0.0145	1.449	24	0.0533	5.333	8.34*E* − 03	1.33*E* − 01	0.261	0.0896–0.7605
C∗03:03:02	3	0.0109	1.087	0						
C∗03:06	3	0.0109	1.087	5	0.0111	1.111				
C∗14:02:01	3	0.0109	1.087	4	0.0089	0.889				
C∗03:02:02	2	0.0072	0.725	0						
C∗02:02:01	1	0.0036	0.362	10	0.0222	2.222				
C∗03:07	1	0.0036	0.362	0						
C∗05:01:02	1	0.0036	0.362	0						
C∗07:04:01	1	0.0036	0.362	6	0.0133	1.333				
C∗17:01:01	1	0.0036	0.362	6	0.0133	1.333				
C∗18:01	1	0.0036	0.362	0						
C∗03:05:01	0			1	0.0022	0.222				
C∗07:01:03	0			1	0.0022	0.222				

INF-P: influenza patients; INF-C: influenza contacts; HLA: human leukocyte antigen; AF: allele frequency; *n*: number of subjects; OR: odds ratio; CI: confidence interval; pC: *p* value after Bonferroni correction; only showing *p* value < 0.05, which is considered significant.

**Table 5:** HLA-A-B-C, A-B, B-C, and A-C haplotype frequencies in patients with influenza A/H1N1 infection (INF-P) and asymptomatic healthy contacts (INF-C).

Haplotype	INF-P *n* = 138		INF-C *n* = 225		*p*	pC	OR	95% CI
	HF	%	HF	%				
A∗02:01:01-B∗35:01:01-C∗04:01:01	0.004	0.36	0.026	2.57	2.19*E* − 02	2.19*E* − 01	0.13	0.01–0.91
A∗24:02:01-B∗35:01:01	0.012	1.23	0.046	4.59	7.99*E* − 03	1.04*E* − 01	0.22	0.06–0.74
B∗35:01:01-C∗07:02:01	0.035	3.54	0.002	0.23	4.15*E* − 04	6.64*E* − 03	16.88	2.14–132.60
B∗39:01:01-C∗07:02:01	0.006	0.63	0.042	4.22	5.35*E* − 03	8.56*E* − 02	0.17	0.04–0.71
B∗40:02:01-C∗03:04:01	0.004	0.36	0.042	4.21	1.56*E* − 03	2.50*E* − 02	0.08	0.01–0.62
A∗68:01:02-C∗07:02:01	0.05	4.96	0.002	0.22	1.05*E* − 05	2.63*E* − 04	23.99	3.14–183.51

Haplotypes with uncorrected *p* value < 0.05, which is considered significant, are only presented. All *p* values were obtained by Fisher exact two-tailed test. INF-P: influenza patients; INF-C: influenza contacts; HF: haplotype frequency; *n*: number of subjects; OR: odds ratio; CI: confidence interval; pC: *p* value after Bonferroni correction. For a complete list of haplotypes, check Tables SIX–SXII.

## Supplementary Materials

Supplementary Materials Table SVI: Complete list of HLA-A alleles in Mexican mestizo population with and without infection by influenza A/H1N1 virus. Table SVII: Complete list of HLA-B alleles in Mexican mestizo population with and without infection by influenza A/H1N1 virus. Table SVIII: Complete list of HLA-C alleles in Mexican mestizo population with and without infection by influenza A/H1N1 virus. Table SIX: HLA-A-B-C haplotype frequencies in patients with influenza A/H1N1 infection and asymptomatic healthy contacts. Table SX: HLA-A-B haplotype frequencies in patients with influenza A/H1N1 infection and asymptomatic healthy contacts. Table SXI: HLA-B-C haplotype frequencies in patients with influenza A/H1N1 infection and asymptomatic healthy contacts. Table SXII: HLA-A-C haplotype frequencies in patients with influenza A/H1N1 infection and asymptomatic healthy contacts.
