# Supplementary material for: An Increased Frequency in HLA Class I Alleles and Haplotypes Suggests Genetic Susceptibility to Influenza A (H1N1) 2009 Pandemic: A Case-Control Study
Source: J Immunol Res. 2018 Feb 25;2018:3174868. doi: 10.1155/2018/3174868 (PMC5845504; doi:10.1155/2018/3174868)
Supplement: Supplementary Materials — Table SVI: Complete list of HLA-A alleles in Mexican mestizo population with and without infection by influenza A/H1N1 virus. Table SVII: Complete list of HLA-B alleles in Mexican mestizo population with and without infection by influenza A/H1N1 virus. Table SVIII: Complete list of HLA-C alleles in Mexican mestizo population with and without infection by influenza A/H1N1 virus. Table SIX: HLA-A-B-C haplotype frequencies in patients with influenza A/H1N1 infection and asymptomatic healthy contacts. Table SX: HLA-A-B haplotype frequencies in patients with influenza A/H1N1 infection and asymptomatic healthy contacts. Table SXI: HLA-B-C haplotype frequencies in patients with influenza A/H1N1 infection and asymptomatic healthy contacts. Table SXII: HLA-A-C haplotype frequencies in patients with influenza A/H1N1 infection and asymptomatic healthy contacts. [file 3174868.f1.docx]

**Supplementary information**

Table VI. Complete list about *HLA-A* alleles in Mexican mestizo population with and without infection by influenza A/H1N1 virus.

| Allele | INF-P n=138 | | |  | INF-C n=225 | | |
| --- | --- | --- | --- | --- | --- | --- | --- |
|  | n | AF | (%) |  | n | AF | (%) |
| A*02:01:01 | 69 | 0.250000 | 25.000 |  | 121 | 0.268889 | 26.889 |
| A*68:01:02 | 32 | 0.115942 | 11.594 |  | 41 | 0.091111 | 9.111 |
| A*03:0101 | 21 | 0.076087 | 7.609 |  | 27 | 0.060000 | 6.000 |
| A*24:02:01 | 20 | 0.072464 | 7.246 |  | 81 | 0.180000 | 18.000 |
| A*31:01:02 | 20 | 0.072464 | 7.246 |  | 23 | 0.051111 | 5.111 |
| A*01:01:01 | 18 | 0.065217 | 6.522 |  | 23 | 0.051111 | 5.111 |
| A*02:06:01 | 17 | 0.061594 | 6.159 |  | 19 | 0.042222 | 4.222 |
| A*29:02:01 | 10 | 0.036232 | 3.623 |  | 10 | 0.022222 | 2.222 |
| A*26:01:01 | 8 | 0.028986 | 2.899 |  | 8 | 0.017778 | 1.778 |
| A*32:01:01 | 8 | 0.028986 | 2.899 |  | 7 | 0.015556 | 1.556 |
| A*30:02:01 | 7 | 0.025362 | 2.536 |  | 10 | 0.022222 | 2.222 |
| A*23:01:01 | 6 | 0.021739 | 2.174 |  | 14 | 0.031111 | 3.111 |
| A*26:01:02 | 5 | 0.018116 | 1.812 |  | 4 | 0.008889 | 0.889 |
| A*68:01:01 | 5 | 0.018116 | 1.812 |  | ND |  |  |
| A*01:03:01 | 3 | 0.010870 | 1.087 |  | 1 | 0.002222 | 0.222 |
| A*11:24:02 | 3 | 0.010870 | 1.087 |  | ND |  |  |
| A*33:01:02 | 3 | 0.010870 | 1.087 |  | 11 | 0.024444 | 2.444 |
| A*66:02:01 | 3 | 0.010870 | 1.087 |  | ND |  |  |
| A*68:02:01 | 3 | 0.010870 | 1.087 |  | 1 | 0.002222 | 0.222 |
| A*24:01:01 | 2 | 0.007246 | 0.725 |  | 3 | 0.006667 | 0.667 |
| A*25:01:01 | 2 | 0.007246 | 0.725 |  | 3 | 0.006667 | 0.667 |
| A*33:01:01 | 2 | 0.007246 | 0.725 |  | ND |  |  |
| A*02:04:01 | 1 | 0.003623 | 0.362 |  | ND |  |  |
| A*02:05:01 | 1 | 0.003623 | 0.362 |  | ND |  |  |
| A*11:01:01 | 1 | 0.003623 | 0.362 |  | 13 | 0.028889 | 2.889 |
| A*23:01:02 | 1 | 0.003623 | 0.362 |  | ND |  |  |
| A*24:02:06 | 1 | 0.003623 | 0.362 |  | ND |  |  |
| A*30:01:01 | 1 | 0.003623 | 0.362 |  | 1 | 0.002222 | 0.222 |
| A*32:02:01 | 1 | 0.003623 | 0.362 |  | 1 | 0.002222 | 0.222 |
| A*68:03:01 | 1 | 0.003623 | 0.362 |  | 1 | 0.002222 | 0.222 |
| A*74:02:01 | 1 | 0.003623 | 0.362 |  | ND |  |  |
| A*01:01:02 | ND |  |  |  | 2 | 0.004444 | 0.444 |
| A*02:01:02 | ND |  |  |  | 1 | 0.002222 | 0.222 |
| A*02:02:01 | ND |  |  |  | 3 | 0.006667 | 0.667 |
| A*02:03:01 | ND |  |  |  | 1 | 0.002222 | 0.222 |
| A*11:01:02 | ND |  |  |  | 1 | 0.002222 | 0.222 |
| A*11:04:01 | ND |  |  |  | 1 | 0.002222 | 0.222 |
| A*26:02:01 | ND |  |  |  | 1 | 0.002222 | 0.222 |
| A*31:02:01 | ND |  |  |  | 4 | 0.008889 | 0.889 |
| A*36:01:01 | ND |  |  |  | 4 | 0.008889 | 0.889 |
| A*66:01:01 | ND |  |  |  | 3 | 0.006667 | 0.667 |
| A*68:01:03 | ND |  |  |  | 1 | 0.002222 | 0.222 |
| A*68:03:02 | ND |  |  |  | 1 | 0.002222 | 0.222 |
| A*68:05:01 | ND |  |  |  | 1 | 0.002222 | 0.222 |
| A*69:01:01 | ND |  |  |  | 1 | 0.002222 | 0.222 |
| A*74:01:01 | ND |  |  |  | 2 | 0.004444 | 0.444 |

ND: Not detected.

Table VII. Complete list about *HLA-B* alleles in Mexican mestizo population with and without infection by influenza A/H1N1 virus.

| Allele | INF-P n=138 | | |  | INF-C n=225 | | |
| --- | --- | --- | --- | --- | --- | --- | --- |
|  | n | AF | (%) |  | n | AF | (%) |
| B*35:01:01 | 37 | 0.134058 | 13.406 |  | 66 | 0.146666 | 14.667 |
| B*15:01:01 | 25 | 0.090580 | 9.058 |  | 33 | 0.073333 | 7.333 |
| B*07:02:01 | 17 | 0.061594 | 6.159 |  | 25 | 0.055556 | 5.556 |
| B*48:01:01 | 16 | 0.057971 | 5.797 |  | 19 | 0.042222 | 4.222 |
| B*39:06:02 | 15 | 0.054348 | 5.435 |  | 10 | 0.022222 | 2.222 |
| B*40:02:01 | 14 | 0.050725 | 5.073 |  | 26 | 0.057778 | 5.778 |
| B*51:01:05 | 11 | 0.039855 | 3.986 |  | 3 | 0.006667 | 0.667 |
| B*44:03:01 | 10 | 0.036232 | 3.623 |  | 3 | 0.006667 | 0.667 |
| B*52:01:02 | 10 | 0.036232 | 3.623 |  | 15 | 0.033333 | 3.333 |
| B*39:05:01 | 9 | 0.032609 | 3.261 |  | 8 | 0.017778 | 1.778 |
| B*51:01:01 | 9 | 0.032609 | 3.261 |  | 32 | 0.071111 | 7.111 |
| B*08:01:01 | 8 | 0.028986 | 2.899 |  | 10 | 0.022222 | 2.222 |
| B*14:02:01 | 7 | 0.025362 | 2.536 |  | 11 | 0.024444 | 2.444 |
| B*58:01:01 | 6 | 0.021739 | 2.174 |  | 3 | 0.006667 | 0.667 |
| B*73:01:01 | 6 | 0.021739 | 2.174 |  | 1 | 0.002222 | 0.222 |
| B*13:02:01 | 5 | 0.018116 | 1.812 |  | 4 | 0.008889 | 0.889 |
| B*18:01:01 | 5 | 0.018116 | 1.812 |  | 9 | 0.020000 | 2.000 |
| B*35:17:01 | 5 | 0.018116 | 1.812 |  | 4 | 0.008889 | 0.889 |
| B*38:01:01 | 4 | 0.014493 | 1.449 |  | ND |  |  |
| B*39:01:01 | 4 | 0.014493 | 1.449 |  | 36 | 0.080000 | 8.000 |
| B*40:05:01 | 4 | 0.014493 | 1.449 |  | 1 | 0.002222 | 0.222 |
| B*44:02:01 | 4 | 0.014493 | 1.449 |  | 16 | 0.035556 | 3.556 |
| B*49:01:01 | 4 | 0.014493 | 1.449 |  | 6 | 0.013333 | 1.333 |
| B*14:06:02 | 3 | 0.010870 | 1.087 |  | ND |  |  |
| B*15:03:01 | 3 | 0.010870 | 1.087 |  | 5 | 0.011111 | 1.111 |
| B*35:02:01 | 3 | 0.010870 | 1.087 |  | 6 | 0.013333 | 1.333 |
| B*39:06:01 | 3 | 0.010870 | 1.087 |  | 6 | 0.013333 | 1.333 |
| B*40:01:01 | 3 | 0.010870 | 1.087 |  | 6 | 0.013333 | 1.333 |
| B*14:01:01 | 2 | 0.007246 | 0.725 |  | 3 | 0.006667 | 0.667 |
| B*27:03:01 | 2 | 0.007246 | 0.725 |  | 6 | 0.013333 | 1.333 |
| B*27:05:02 | 2 | 0.007246 | 0.725 |  | 2 | 0.004444 | 0.444 |
| B*35:05:01 | 2 | 0.007246 | 0.725 |  | ND |  |  |
| B*35:12:01 | 2 | 0.007246 | 0.725 |  | 9 | 0.020000 | 2.000 |
| B*39:02:02 | 2 | 0.007246 | 0.725 |  | 3 | 0.006667 | 0.667 |
| B*41:01:01 | 2 | 0.007246 | 0.725 |  | 4 | 0.008889 | 0.889 |
| B*53:01:01 | 2 | 0.007246 | 0.725 |  | 2 | 0.004444 | 0.444 |
| B*15:02:01 | 1 | 0.003623 | 0.362 |  | 4 | 0.008889 | 0.889 |
| B*15:16:01 | 1 | 0.003623 | 0.362 |  | ND |  |  |
| B*35:01:02 | 1 | 0.003623 | 0.362 |  | ND |  |  |
| B*45:01:01 | 1 | 0.003623 | 0.362 |  | 5 | 0.011111 | 1.111 |
| B*57:01:01 | 1 | 0.003623 | 0.362 |  | 6 | 0.013333 | 1.333 |
| B*78:02:02 | 1 | 0.003623 | 0.362 |  | ND |  |  |
| B*13:01:01 | ND |  |  |  | 1 | 0.002222 | 0.222 |
| B*15:05:01 | ND |  |  |  | 1 | 0.002222 | 0.222 |
| B*15:15:01 | ND |  |  |  | 2 | 0.004444 | 0.444 |
| B*35:01:04 | ND |  |  |  | 2 | 0.004444 | 0.444 |
| B*37:01:01 | ND |  |  |  | 1 | 0.002222 | 0.222 |
| B*38:01:01 | ND |  |  |  | 6 | 0.013333 | 1.333 |
| B*38:01:02 | ND |  |  |  | 6 | 0.013333 | 1.333 |
| B*40:01:02 | ND |  |  |  | 1 | 0.002222 | 0.222 |
| B*40:01:04 | ND |  |  |  | 3 | 0.006667 | 0.667 |
| B*40:02:03 | ND |  |  |  | 1 | 0.002222 | 0.222 |
| B*40:11:01 | ND |  |  |  | 1 | 0.002222 | 0.222 |
| B*42:01:01 | ND |  |  |  | 2 | 0.004444 | 0.444 |
| B*45:01:03 | ND |  |  |  | 1 | 0.002222 | 0.222 |
| B*46:02:01 | ND |  |  |  | 1 | 0.002222 | 0.222 |
| B*50:01:01 | ND |  |  |  | 3 | 0.006667 | 0.667 |
| B*51:02:01 | ND |  |  |  | 1 | 0.002222 | 0.222 |
| B*55:01:01 | ND |  |  |  | 3 | 0.006667 | 0.667 |
| B*55:02:01 | ND |  |  |  | 2 | 0.004444 | 0.444 |
| B*56:01:01 | ND |  |  |  | 1 | 0.002222 | 0.222 |
| Not determined | 4 | 0.014493 | 1.449 |  | 3 | 0.006667 | 0.667 |

ND: Not detected.

Table VIII. Complete list about *HLA-C* alleles in Mexican mestizo population with and without infection by influenza A/H1N1 virus.

| Allele | INF-P n=138 | | |  | INF-C n=225 | | |
| --- | --- | --- | --- | --- | --- | --- | --- |
|  | n | AF | (%) |  | n | AF | (%) |
| C*07:02:01 | 61 | 0.221014 | 22.101 |  | 60 | 0.133333 | 13.333 |
| C*04:01:01 | 50 | 0.181159 | 18.116 |  | 74 | 0.164444 | 16.444 |
| C*01:02:01 | 22 | 0.079710 | 7.971 |  | 31 | 0.068889 | 6.889 |
| C*08:01:01 | 20 | 0.072464 | 7.246 |  | 26 | 0.057778 | 5.778 |
| C*07:01:01 | 13 | 0.047101 | 4.710 |  | 42 | 0.093333 | 9.333 |
| C*03:04:01 | 12 | 0.043478 | 4.348 |  | 48 | 0.106667 | 10.667 |
| C*08:02:01 | 11 | 0.039855 | 3.986 |  | 15 | 0.033333 | 3.333 |
| C*03:02:01 | 10 | 0.036232 | 3.623 |  | 5 | 0.011111 | 1.111 |
| C*06:02:01 | 10 | 0.036232 | 3.623 |  | 13 | 0.028889 | 2.889 |
| C*16:01:01 | 10 | 0.036232 | 3.623 |  | 17 | 0.037778 | 3.778 |
| C*05:01:01 | 8 | 0.028986 | 2.899 |  | 19 | 0.042222 | 4.222 |
| C*12:03:01 | 6 | 0.021739 | 2.174 |  | 22 | 0.048889 | 4.889 |
| C*03:01:01 | 5 | 0.018116 | 1.812 |  | 1 | 0.002222 | 0.222 |
| C*08:01:02 | 5 | 0.018116 | 1.812 |  | ND |  |  |
| C*15:02:01 | 5 | 0.018116 | 1.812 |  | 21 | 0.046667 | 4.667 |
| C*02:01:01 | 4 | 0.014493 | 1.449 |  | ND |  |  |
| C*03:03:01 | 3 | 0.010870 | 1.087 |  | 23 | 0.051111 | 5.111 |
| C*03:03:02 | 3 | 0.010870 | 1.087 |  | ND |  |  |
| C*03:06:01 | 3 | 0.010870 | 1.087 |  | 5 | 0.011111 | 1.111 |
| C*02:02:02 | 2 | 0.007246 | 0.725 |  | ND |  |  |
| C*03:02:02 | 2 | 0.007246 | 0.725 |  | ND |  |  |
| C*12:01:01 | 2 | 0.007246 | 0.725 |  | ND |  |  |
| C*14:01:01 | 2 | 0.007246 | 0.725 |  | ND |  |  |
| C*02:02:01 | 1 | 0.003623 | 0.362 |  | 10 | 0.022222 | 2.222 |
| C*03:07:01 | 1 | 0.003623 | 0.362 |  | ND |  |  |
| C*05:01:02 | 1 | 0.003623 | 0.362 |  | ND |  |  |
| C*07:04:01 | 1 | 0.003623 | 0.362 |  | 6 | 0.013333 | 1.333 |
| C*14:02:01 | 1 | 0.003623 | 0.362 |  | 4 | 0.008889 | 0.889 |
| C*17:01:01 | 1 | 0.003623 | 0.362 |  | 6 | 0.013333 | 1.333 |
| C*18:01:01 | 1 | 0.003623 | 0.362 |  | ND |  |  |
| C*03:05:01 | ND |  |  |  | 1 | 0.002222 | 0.222 |
| C*07:01:03 | ND |  |  |  | 1 | 0.002222 | 0.222 |

ND: Not detected.

Table IX. HLA A-B-C haplotype frequencies in patients with influenza A/H1N1 infection and asymptomatic healthy contacts.

| Haplotype A-B-C | INF-P  n=138 HF | INF-C  n=225 HF | p | OR | CI (95%) |
| --- | --- | --- | --- | --- | --- |
| A*03:01:01-B*35:01:01-C*04:01:01 | 0.014493 | 0.002628 |  |  |  |
| A*24:02:01-B*15:01:01-C*01:02:01 | 0.014493 | 0.002709 |  |  |  |
| A*68:01:02-B*35:01:01-C*04:01:01 | 0.014493 | 0.002288 |  |  |  |
| A*02:01:01-B*15:01:01-C*01:02:01 | 0.010870 | 0.015068 |  |  |  |
| A*02:01:01-B*48:01:01-C*04:01:01 | 0.010870 | 0.004444 |  |  |  |
| A*03:01:01-B*07:02:01-C*07:02:01 | 0.010870 | 0.013333 |  |  |  |
| A*02:01:01-B*40:01:01-C*03:04:01 | 0.007246 | 0.002222 |  |  |  |
| A*02:06:01-B*35:01:01-C*04:01:01 | 0.007246 | 0.004444 |  |  |  |
| A*24:02:01-B*07:02:01-C*07:02:01 | 0.007246 | 0.004444 |  |  |  |
| A*68:01:02-B*39:05:01-C*07:02:01 | 0.007246 | 0.002222 |  |  |  |
| A*02:01:01-B*35:01:01-C*04:01:01 | 0.003728 | 0.029429 | 0.02231 | 0.122 | 0.02-0.94 |
| A*01:01:01-B*13:02:01-C*07:02:01 | 0.003623 | 0.002222 |  |  |  |
| A*02:01:01-B*35:02:01-C*03:01:01 | 0.003623 | 0.002222 |  |  |  |
| A*02:06:01-B*48:01:01-C*07:02:01 | 0.003623 | 0.002222 |  |  |  |
| A*03:01:01-B*13:02:01-C*06:02:01 | 0.003623 | 0.002222 |  |  |  |
| A*24:02:01-B*51:01:01-C*04:01:01 | 0.003623 | 0.002461 |  |  |  |
| A*31:01:02-B*51:01:05-C*04:01:01 | 0.003623 | 0.002222 |  |  |  |
| A*32:01:01-B*14:01:01-C*08:02:01 | 0.003623 | 0.002222 |  |  |  |
| A*33:01:02-B*35:12:01-C*04:01:01 | 0.003623 | 0.002222 |  |  |  |
| A*68:01:02-B*15:01:01-C*01:02:01 | 0.003623 | 0.006667 |  |  |  |
| A*68:01:02-B*51:01:01-C*08:01:01 | 0.003623 | 0.004444 |  |  |  |
| ^#^A*02:01:01-B*39:01:01-C*07:02:01 | ND | 0.024444 | 0.00867 | 0.00 | Und |
| ^#^A*24:02:01-B*35:01:01-C*03:04:01 | ND | 0.019871 | 0.01555 | 0.00 | Und |
| ^#^A*24:02:01-B*35:01:01-C*04:01:01 | ND | 0.025384 | 0.00867 | 0.00 | Und |

Only extended haplotypes present in both study groups are shown.

# Exceptions. ND: Not detected. Und: Undefined.

Table X. HLA A-B haplotype frequencies in patients with influenza A/H1N1 infection and asymptomatic healthy contacts.

| Haplotype A-B | INF-P  n=138 HF | INF-C  n=225 HF | p | OR | CI (95%) |
| --- | --- | --- | --- | --- | --- |
| A*24:02:01-B*35:01:01 | 0.005891 | 0.044680 | 0.003287 | 0.16 | 0.04-0.68 |
| A*02:01:01-B*48:01:01 | 0.020501 | 0.004298 |  |  |  |
| A*24:02:01-B*39:01:01 | 0.003623 | 0.021185 |  |  |  |
| A*02:01:01-B*39:01:01 | 0.007246 | 0.027834 |  |  |  |
| A*02:01:01-B*51:01:01 | 0.010870 | 0.033862 |  |  |  |
| A*31:01:02-B*48:01:01 | 0.009250 | 0.002740 |  |  |  |
| A*02:01:01-B*39:05:01 | 0.017236 | 0.006667 |  |  |  |
| A*24:02:01-B*40:02:01 | 0.007246 | 0.018971 |  |  |  |
| A*02:06:01-B*35:01:01 | 0.004466 | 0.012265 |  |  |  |
| A*02:01:01-B*44:02:01 | 0.014493 | 0.006667 |  |  |  |
| A*02:01:01-B*39:06:02 | 0.014493 | 0.008655 |  |  |  |
| A*31:01:02-B*40:02:01 | 0.007246 | 0.014772 |  |  |  |
| A*30:02:01-B*35:01:01 | 0.003623 | 0.004444 |  |  |  |
| A*01:01:01-B*58:01:01 | 0.007246 | 0.001906 |  |  |  |
| A*03:01:01-B*49:01:01 | 0.007246 | 0.002222 |  |  |  |
| A*02:01:01-B*52:01:02 | 0.028986 | 0.022222 |  |  |  |
| A*02:01:01-B*14:02:01 | 0.007246 | 0.003617 |  |  |  |
| A*02:01:01-B*40:02:01 | 0.008265 | 0.005241 |  |  |  |
| A*31:01:02-B*35:01:01 | 0.010870 | 0.007463 |  |  |  |
| A*02:01:01-B*15:01:01 | 0.025040 | 0.033553 |  |  |  |
| A*24:02:01-B*15:01:01 | 0.008602 | 0.011508 |  |  |  |
| A*02:01:01-B*18:01:01 | 0.010870 | 0.015556 |  |  |  |
| A*03:01:01-B*35:01:01 | 0.010870 | 0.015312 |  |  |  |
| A*02:01:01-B*35:01:01- | 0.037653 | 0.038254 |  |  |  |
| A*01:01:01-B*08:01:01 | 0.014493 | 0.013021 |  |  |  |
| A*01:01:01-B*13:02:01 | 0.003623 | 0.002222 |  |  |  |
| A*01:01:01-B*57:01:01 | 0.003623 | 0.002222 |  |  |  |
| A*02:06:01-B*48:01:01- | 0.008023 | 0.007735 |  |  |  |
| A*03:01:01-B*13:02:01 | 0.003623 | 0.002222 |  |  |  |
| A*03:01:01-B*15:01:01 | 0.005166 | 0.00450 |  |  |  |
| A*24:01:01-B*35:01:01 | 0.003623 | 0.002222 |  |  |  |
| A*24:02:01-B*07:02:01 | 0.007246 | 0.009332 |  |  |  |
| A*24:02:01-B*27:03:01 | 0.003623 | 0.006667 |  |  |  |
| A*24:02:01-B*49:01:01 | 0.003623 | 0.002755 |  |  |  |
| A*31:01:02-B*14:02:01 | 0.010870 | 0.002222 |  |  |  |
| A*31:01:02-B*15:03:01 | 0.003623 | 0.002222 |  |  |  |
| A*31:01:02-B*39:06:02 | 0.005242 | 0.002222 |  |  |  |
| A*32:02:01-B*07:02:01 | 0.003623 | 0.002222 |  |  |  |
| A*33:01:02-B*35:12:01 | 0.003623 | 0.002222 |  |  |  |
| A*68:01:02-B*39:05:01 | 0.004503 | 0.002222 |  |  |  |
| A*68:01:02-B*48:01:01 | 0.010870 | 0.010366 |  |  |  |
| A*68:01:02-B*53:01:01 | 0.003623 | 0.002222 |  |  |  |
| A*68:03:01-B*73:01:01 | 0.003623 | 0.002222 |  |  |  |
| A*01:01:01-B*40:02:01 | 0.003623 | 0.000519 |  |  |  |
| ^#^A*68:01:02-B*51:01:05 | 0.020678 | ND | 0.002918 | Und | Und |
| ^#^A*02:01:01-B*35:17:01 | 0.018116 | ND | 0.007763 | Und | Und |

Only haplotypes present in both study groups are shown.

# Exceptions. ND: Not detected. Und: Undefined

Table XI. HLA B-C haplotype frequencies in patients with influenza A/H1N1 infection and asymptomatic healthy contacts.

| Haplotype B-C | INF-P  n=138 HF | INF-C  n=225 HF | p | OR | CI (95%) |
| --- | --- | --- | --- | --- | --- |
| B*40:02:01-C*03:04:01 | 0.003623 | 0.042142 | 0.001564 | 0.08 | 0.01-0.62 |
| B*15:01:01-C*01:02:01 | 0.034784 | 0.027983 | 0.022312 | 0.12 | 0.02-0.94 |
| B*52:01:02-C*03:03:01 | 0.003623 | 0.024424 |  |  |  |
| B*35:01:01-C*03:04:01 | 0.003623 | 0.0218 |  |  |  |
| B*48:01:01-C*04:01:01 | 0.014493 | 0.002222 |  |  |  |
| B*51:01:05-C*04:01:01 | 0.012671 | 0.002222 |  |  |  |
| B*13:02:01-C*07:02:01 | 0.01087 | 0.002222 |  |  |  |
| B*52:01:02-C*03:04:01 | 0.01087 | 0.002243 |  |  |  |
| B*35:12:01-C*04:01:01 | 0.003623 | 0.015556 |  |  |  |
| B*07:02:01-C*04:01:01 | 0.01087 | 0.004655 |  |  |  |
| B*58:01:01-C*07:01:01 | 0.01087 | 0.003963 |  |  |  |
| B*35:01:01-C*04:01:01 | 0.063964 | 0.081728 |  |  |  |
| B*39:05:01-C*07:02:01 | 0.016304 | 0.011111 |  |  |  |
| B*14:02:01-C*08:02:01 | 0.01087 | 0.02 |  |  |  |
| B*13:02:01-C*06:02:01 | 0.007246 | 0.002222 |  |  |  |
| B*35:01:01-C*12:03:01 | 0.007246 | 0.002751 |  |  |  |
| B*15:01:01-C*04:01:01 | 0.01087 | 0.006716 |  |  |  |
| B*44:02:01-C*16:01:01 | 0.01087 | 0.00743 |  |  |  |
| B*51:01:01-C*08:01:01 | 0.01087 | 0.006667 |  |  |  |
| B*07:02:01-C*07:02:01 | 0.028986 | 0.033333 |  |  |  |
| B*48:01:01-C*08:01:01 | 0.026812 | 0.031077 |  |  |  |
| B*39:06:02-C*07:02:01 | 0.014198 | 0.015556 |  |  |  |
| B*39:06:01-C*07:02:01 | 0.01087 | 0.011111 |  |  |  |
| B*40:01:01-C*03:04:01 | 0.007246 | 0.006667 |  |  |  |
| B*51:01:01-C*12:03:01 | 0.007246 | 0.00836 |  |  |  |
| B*07:02:01-C*03:02:02 | 0.003623 | 0.002222 |  |  |  |
| B*15:01:01-C*08:01:01 | 0.004777 | 0.002222 |  |  |  |
| B*15:01:01-C*14:02:01 | 0.003623 | 0.003078 |  |  |  |
| B*27:03:01-C*01:02:01 | 0.003623 | 0.001111 |  |  |  |
| B*30:01:01-C*08:01:01 | 0.003623 | 0.002222 |  |  |  |
| B*38:01:01-C*03:02:01 | 0.003623 | 0.002222 |  |  |  |
| B*39:01:01-C*04:01:01 | 0.003623 | 0.002432 |  |  |  |
| B*39:01:01-C*07:01:01 | 0.003623 | 0.002012 |  |  |  |
| B*39:02:02-C*03:04:01 | 0.003623 | 0.002222 |  |  |  |
| B*39:06:02-C*01:02:01 | 0.003918 | 0.002222 |  |  |  |
| B*39:06:02-C*15:02:01 | 0.003623 | 0.002222 |  |  |  |
| B*44:03:01-C*01:02:01 | 0.003623 | 0.002222 |  |  |  |
| B*53:01:01-C*04:01:01 | 0.003623 | 0.002222 |  |  |  |
| B*57:01:01-C*07:01:01 | 0.003623 | 0.002222 |  |  |  |
| B*15:01:01-C*03:04:01 | 0.003623 | 0.004444 |  |  |  |
| B*15:01:01-C*07:01:01 | 0.003623 | 0.004764 |  |  |  |
| B*15:02:01-C*01:02:01 | 0.003623 | 0.004444 |  |  |  |
| B*35:17:01-C*04:01:01 | 0.003623 | 0.004444 |  |  |  |
| B*39:02:02-C*07:02:01 | 0.003623 | 0.004444 |  |  |  |
| B*45:01:01-C*06:02:01 | 0.003623 | 0.004444 |  |  |  |
| B*52:01:02-C*12:03:01 | 0.003623 | 0.004444 |  |  |  |
| B*14:01:01-C*08:02:01 | 0.003623 | 0.006667 |  |  |  |
| B*40:02:01-C*03:06:01 | 0.001905 | 0.006667 |  |  |  |
| B*35:01:01-C*07:02:01 | 0.045280 | ND | 0.000002896 | Und | Und |

Only haplotypes present in both study groups are shown.

# Exceptions. ND: Not detected. Und: Undefined.

Table XII. HLA A-C haplotype frequencies in patients with influenza A/H1N1 infection and asymptomatic healthy contacts.

| Haplotype A-C | INF-P  n=138 HF | INF-C  n=225 HF | p | OR | CI (95%) |
| --- | --- | --- | --- | --- | --- |
| A*68:01:02-C*07:02:01- | 0.048299 | 0.002222 | 0.0000105 | 23.99 | 3.13-183.50 |
| A*02:01:01-C*05.01:01 | 0.005321 | 0.022222 |  |  |  |
| A*24:02:01-C*07:02:01 | 0.013118 | 0.036953 |  |  |  |
| A*02:06:01-C*08:01:01 | 0.024591 | 0.009610 |  |  |  |
| A*02:01:01-C*03:04:01 | 0.006293 | 0.023751 |  |  |  |
| A*01:01:01-C*08:02:01 | 0.01087 | 0.002222 |  |  |  |
| A*02:01:01-C*03:02:01 | 0.011893 | 0.002844 |  |  |  |
| A*03:01:01-C*06:02:01 | 0.010870 | 0.002280 |  |  |  |
| A*31:01:02-C*03:02:01 | 0.010870 | 0.002222 |  |  |  |
| A*02:01:01-C*01:02:01 | 0.042755 | 0.021848 |  |  |  |
| A*02:01:01-C*07:01:01 | 0.003623 | 0.016729 |  |  |  |
| A*02:01:01-C*08:02:01 | 0.012693 | 0.005258 |  |  |  |
| A*68:01:02-C*08:01:01 | 0.014855 | 0.005212 |  |  |  |
| A*24:02:01-C*07:01:01 | 0.003623 | 0.012807 |  |  |  |
| A*02:01:01-C*07:02:01 | 0.055849 | 0.036474 |  |  |  |
| A*01:01:01-C*07:02:01 | 0.021739 | 0.010745 |  |  |  |
| A*31:01:02-C*07:02:01 | 0.019888 | 0.012176 |  |  |  |
| A*03:01:01-C*01:02:01 | 0.007246 | 0.002822 |  |  |  |
| A*24:02:01-C*04:01:01 | 0.00838 | 0.00397 |  |  |  |
| A*26:01:01-C*12:03:01 | 0.007246 | 0.003616 |  |  |  |
| A*29:02:01-C*16:01:01 | 0.007128 | 0.004444 |  |  |  |
| A*68:01:02-C*03:01:01 | 0.007246 | 0.005202 |  |  |  |
| A*31:01:02-C*01:02:01 | 0.004441 | 0.007816 |  |  |  |
| A*02:01:01-C*08:01:01 | 0.012040 | 0.005585 |  |  |  |
| A*31:01:02-C*04:01:01 | 0.008279 | 0.010471 |  |  |  |
| A*03:01:01-C*04:01:01 | 0.014493 | 0.011718 |  |  |  |
| A*02:06:01-C*07:02:01 | 0.018867 | 0.015363 |  |  |  |
| A*68:01:02-C*03:04:01 | 0.018181 | 0.013799 |  |  |  |
| A*68:01:02-C*04:01:01 | 0.022869 | 0.021302 |  |  |  |
| A*02:01:01-C*16:01:01 | 0.021858 | 0.016710 |  |  |  |
| A*01:01:01-C*07:01:01 | 0.018116 | 0.022222 |  |  |  |
| A*02:01:01-C*04:01:01 | 0.058339 | 0.060361 |  |  |  |
| A*01:01:01-C*03:02:02 | 0.003623 | 0.002222 |  |  |  |
| A*02:06:01-C*07:01:01 | 0.006378 | 0.004444 |  |  |  |
| A*03:01:01-C*02:02:01 | 0.003623 | 0.004444 |  |  |  |
| A*03:01:01-C*08:02:01 | 0.003623 | 0.006667 |  |  |  |
| A*23:01:01-C*04:01:01 | 0.003623 | 0.002222 |  |  |  |
| A*23:01:01-C*08:01:01 | 0.006485 | 0.005855 |  |  |  |
| A*24:02:01-C*01:02:01 | 0.010775 | 0.011294 |  |  |  |
| A*24:02:01-C*08:01:01 | 0.010870 | 0.011812 |  |  |  |
| A*24:02:01-C*12:03:01 | 0.003623 | 0.002280 |  |  |  |
| A*25:01:01-C*08:01:02 | 0.003623 | 0.002222 |  |  |  |
| A*26:01:01-C*16:01:01 | 0.003623 | 0.004444 |  |  |  |
| A*30:02:01-C*04:01:01 | 0.015947 | 0.014236 |  |  |  |
| A*30:02:01-C*07:01:01 | 0.003623 | 0.005764 |  |  |  |
| A*32:01:01-C*12:03:01 | 0.003623 | 0.006667 |  |  |  |
| A*32:02:01-C*04:01:01 | 0.003623 | 0.002222 |  |  |  |
| A*33:01:02-C*08:02:01 | 0.003623 | 0.006667 |  |  |  |
| A*68:01:02-C*07:01:01 | 0.004491 | 0.003267 |  |  |  |
| ^#^A*24:02:01-C*03:04:01 | ND | 0.049567 | 0.0000622 | 0 | Und |
| ^#^A*02:01:01-C*03:03:01 | ND | 0.032575 | 0.0008313 | 0 | Und |

Only haplotypes present in both study groups are shown.

# Exceptions. ND: Not detected. Und: Undefined.
